# A fast and stable vascular deformation scheme for interventional surgery training system

**DOI:** 10.1186/s12938-016-0148-3

**Published:** 2016-04-06

**Authors:** Xiufen Ye, Jianguo Zhang, Peng Li, Tian Wang, Shuxiang Guo

**Affiliations:** College of Automation, Harbin Engineering University, No. 145, Nantong street, Harbin, China; Intelligent Mechanical Systems Engineering, Kagawa University, Hayashi-cho, Takamatsu, 761-0396 Japan

**Keywords:** Vascular interventional surgery, Surgery training, Position-based dynamic, Volume conservation, Spatial acceleration

## Abstract

**Background:**

The emergence and development of robot assistant interventional vascular surgery technologies have benefited many patients with cardiovascular or cerebrovascular diseases. Due to the absence of effective training measures, these new advanced technologies have not been fully utilized and only few experienced surgeons can perform such complicated surgeries so far. In order to solve such problems, virtual reality based vascular interventional surgery training system, a promising way to train young surgeons or assist experienced surgeons to perform surgery, has been widely studied.

**Methods:**

In this paper, we mainly conduct a thorough study on both reliable deformation and high real-time performance of an interactive surgery training system. An efficient hybrid geometric blood vessel model which handles the collision detection query and vascular deformation calculation separately is employed to enhance the real-time performance of our surgery training system. In addition, a position-based dynamic approach with volume conservation constraint is used to improve the vascular deformation result. Finally, a hash table based spatial adaptive acceleration algorithm which makes the training system much more efficient and reliable is described.

**Results:**

Several necessary experiments are conducted to validate the vascular deformation scheme presented in this paper. From the results we can see that the position-based dynamic modeling method with volume conservation constraint can prevent the vascular deformation from the issue of penetration. In addition, the deformation calculation with spatial acceleration algorithm has enhanced the real-time performance significantly.

**Conclusion:**

The corresponding experimental results indicate that both the hybrid geometric blood vessel model and the hash table based spatial adaptive acceleration algorithm can enhance the performance of our surgery training system greatly without losing the deformation accuracy.

## Background

**Fig. 1:**
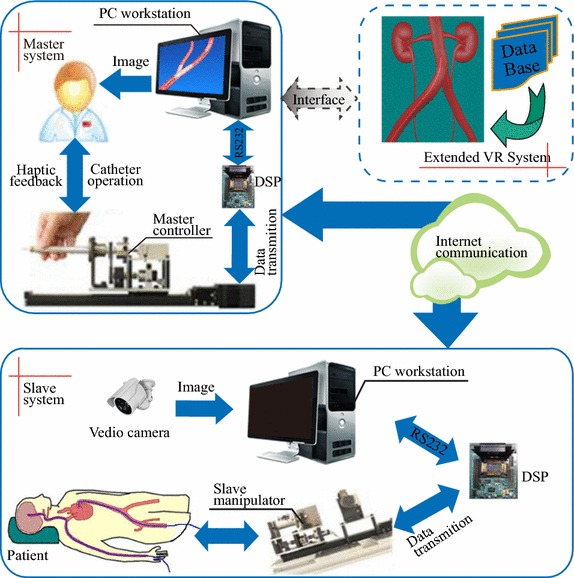
Illustration of a master–slave based robotic assistant vascular interventional surgery system

In vascular interventional surgery especially in current advanced master–slave based vascular interventional surgery, surgeons are required extremely strict professional skills. Only few experienced surgeons can perform such complicated surgery by now mainly due to the difficulty to learn and master the skill as well as the existed gap between engineering development and medical training. Virtual reality based surgery training system is regarded as a promising way to overcome these problems [1–3]. As shown in Fig. 1, a master–slave based robot assistant vascular interventional surgery system usually contains a master system controlled by a surgeon directly, a corresponding slave system and a virtual reality based surgery training system. With years of development, the technique of robotic assistant vascular interventional surgery system has made a great progress, while the relevant surgery training system seems a little incompetent. There are lots of issues that have to be solved or balanced in developing an interactive surgery training system in real-time [4]. For instance, accurate vascular deformation result can be obtained only with a more accurate biomechanics modeling approach which may reduce the real-time performance of the training system. High refresh rate of haptic feedback device, network transmission with minimum time-delay and data loss are also required to guarantee a consecutive and stable force feedback.

There are already several academic or commercial vascular interventional assistant surgery robotic prototypes which have been promoting the development of the minimally invasive surgery. Guo and Guo proposed a novel master–slave based robotic catheter operating system with visual and force feedback [2], but a corresponding surgery training system is urgent to be studied. Wang et al. also designed a master–slave based vascular interventional surgery with an image navigation system, and validated their robotic system with transparent glass vascular model and animal experiments [5, 6]. Meng et al. addressed several key technologies of image guided system, like distortion correction, catheter localization and clinical simulation [7]. Since a vascular surgery training system can be used for training and assisting a surgeon to perform interventional surgery and so on, lots of researchers have shown their great interests in developing surgery training system. Zhang et al. used a mass-spring model to simulate vascular deformation [8], whose spring coefficient is driven from a reference model, and used more accurate finite element method to validate the simulation results. However, the collision detection and its response performance are not given. Wu et al. used flexible elastic rods to model the guidewire in interventional radiology with different resolutions, which are adaptive to the curvature of the vessel dynamically [9], moreover, they also proposed a force correction strategy to decrease the overhead of collision detection and thus improve the computation efficiency significantly [10]. Lenoir et al. used an incremental linear finite element model to approximate the geometric non-linearity of large bending deformation of the catheter. In addition, a substructure analysis is used for improving the computational efficiency [11]. Tang et al. presented a real-time physically based model to simulate catheter insertions, and non-linear elastic cosserat rods is used to simulate the long, slender body of the catheter shaft [12].

In the field of mechanics, many feasible modeling approaches are available for deformable simulation and mainly range from simple and fast mass-spring system (MSS) to complicated but accurate finite element method (FEM). The early MSSs are widely used due to its efficiency and simplicity, and some improvements are well studied to make them more suitable for medical simulation with complicated biomechanics properties in real-time [13–16]. Continuous mechanics based FEMs are much more accurate than MSSs but accompanied with huge computation overhead and not suitable for a real-time interactive surgery training system. Some efficient measures are taken to accelerate the computation, like pre-computation, linear approximation, and GPUs acceleration so as to simulate the deformation with haptic feedback in real-time properly [17]. Physics modeling method used in our surgery training system is a position-based dynamic approach, proposed by Müller et al. [18, 19]. Position-based method is well established and used widely, even in famous physics dynamic libraries like PhysX and Bullet. The main advantages of this approach are its controllability, and the problem of overshooting in force based explicit integration systems can be avoided. All these advanced properties make the approach standout when comparing with above mentioned MSS and FEM in developing a real-time interactive surgery training system. Moreover, position-based dynamic approach is geometry motivated and unconditional stable even with large time step so that this method can guarantee the stability of the surgery training system better than using MSSs which may become unstable when facing large time and the parameters in a mass-spring system are much more arbitrary. General position-based method is often used in computer animation and mainly handles with triangular meshes, while the basic element is tetrahedron in our blood vessel geometric model. Therefore, an extra volume conservation constraint need to be considered in our implementation to guarantee the deformation accuracy.

This paper contributes to a master–slave based vascular interventional surgery training system mainly in three aspects. First, a hybrid blood vessel model was designed to process collision detection and deformation calculation separately to improve the real-time performance of our surgery training system. Second, volume conservation constraint of a tetrahedral element was deduced and then used to solve the problem of tetrahedral elements overlapping which improves the vascular deformation result. Lastly, spatial acceleration algorithm is employed to enhance the real-time performance of our interactive training system.

The remainder of the paper is organized as follows. In "Methods" section, we introduce the hybrid geometric blood vessel modeling approach and the volume conservation constraint of a tetrahedral element, and then a hash table based spatial acceleration algorithm is depicted. Simulation results are given in "Conclusion" section, and finally we discuss our approaches and future work planning systematically.

## Methods

### Vascular deformation modeling scheme

Efficient vascular deformation modeling scheme plays an important role in virtual reality based vascular interventional surgery training system since it determines the visual deformation result and real-time performance directly. In this section, we first discuss a hybrid geometric blood vessel model which processes collision detection and deformation calculation separately, and then introduce a position-based vascular deformation method with volume conservation.

#### Hybrid geometric blood vessel model

**Fig. 2:**
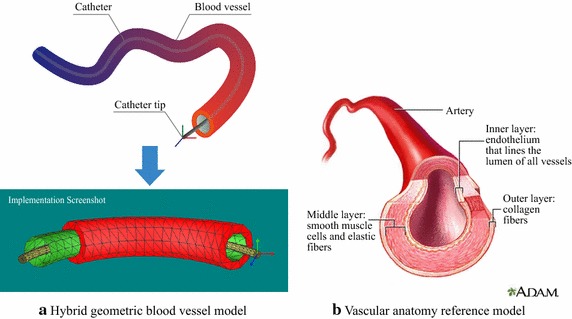
Hybrid geometric blood vessel model and its reference. **a** The hybrid geometric model with *tetrahedral* and *triangular mesh*, and **b** A real vascular anatomy model (from http://www.adam.com) which provides a reference for our hybrid geometric model

A hybrid geometric blood vessel model which consists of a relative simplified triangular mesh and a complicated tetrahedral mesh is employed in this paper to improve the efficiency of collision detection between catheter and vascular wall. Figure 2a depicts the conceptual diagram of the proposed hybrid blood vessel model where the tetrahedral mesh represents vascular wall with specific thickness inspired by the vascular anatomy model, as shown in Fig. 2b. The red tetrahedral mesh is mainly used for accurate deformation calculation, while the green triangular mesh represents the ghost surface (never rendering) that tightly attached to the inner vascular wall. This triangular mesh is mainly designed for collision detection between the catheter and the blood vessel and further preventing the catheter from penetrating the blood vessel. Compared with previous related work [9], employing position-based dynamics technique with volume conservation and hash table based spatial acceleration scheme for real-time vessel deformation simulation is our main concern in this paper. Therefore, we simply model the catheter with tetrahedral mesh (yellow cylinder) mainly for further efficient collision detection and convenient experimental results validation.

The motion states of a flexible catheter and its contact information with vascular wall are also important in this paper. In our implementation, the catheter is also modeled with tetrahedral mesh and its tip has four degrees of freedom (DOF) just as a real catheter, including three degrees of freedom in rotation (i.e. rotation around x, y and z axis) and one translation along the catheter axis direction. The rest parts are controlled by elastic potential energy produced by the collision response (collision between the catheter and the ghost triangular mesh). In this way, a catheter can move freely in the blood vessel with continuous transmission commands governed by an experienced surgeon.

#### Position-based vascular deformation with volume conservation

Compared with the widely-used mass-spring system, position-based dynamic approach is unconditional stable even solved with a large time step. The original studies are mainly used to model the deformation of triangular mesh like cloth simulation [19, 20]. Cloth can be constructed with a large amount of triangles easily and its constraints mainly consist of stretching and bending constraints. As we mentioned above, the elements in our deformation calculation are tetrahedrons rather than triangles so that volume conservation constraint should be also taken into account as well as stretching and bending constraints to prevent the vascular wall from the issue of penetration and makes the deformation much more realistic. However, the volume conservation constraint is just mentioned briefly without detailed derivation in the original study [18]. To implement the position-based vascular deformation with volume conservation, a detailed derivation of the constraint should be given firstly. Let *p* be the vertices concatenation ${\bigl [p_1^T, p_2^T, p_3^T, p_4^T\bigr ]}^{T}$ of a tetrahedron. Given a vertex $p_i$ with mass $m_i$, where $i \in (1,2,3,4)$ is the index number of a vertex, as well as *j* in the following equation. Then, the displacement of the vertex $p_i$ by projection can be represented as

1 $$\begin{aligned} \Delta p_i =-\frac{C(p)}{\sum _{j=1}^4 w_j {|\nabla _{p_j} C(p)|}^{2}} w_i \nabla _{p_i} C(p) \end{aligned}$$

where $w_i=1 / m_i$, *C*(*p*) and $\nabla _p C(p)$ mean the constraint function and its gradient respectively. According to the reference [19], the volume conservation constraint of a single tetrahedron with rest volume $V_0$ can be described as

2 $$\begin{aligned} C(p_1, p_2, p_3, p_4)=\frac{1}{6}\left( (p_2 - p_1) \times (p_3 - p_1)\right) \cdot (p_4 - p_1)- V_0 \end{aligned}$$

And our ultimate goal is to deduce every unknown factor in Eq. (1). The volume of a tetrahedron can be calculated with

3 $$\begin{aligned} V(p_1, p_2, p_3, p_4)&= \frac{1}{6}\left( (p_2 - p_1) \times (p_3 - p_1)\right) \cdot (p_4 - p_1) \nonumber \\&= \frac{1}{6}\left( (p_2 \times p_3 - p_2 \times p_1 - p_1 \times p_3 + p_1 \times p_1)\right) \cdot (p_4 - p_1) \nonumber \\&= \frac{1}{6}\left( (p_2 \times p_3)\cdot p_4 - (p_2 \times p_1)\cdot p_4 - (p_1 \times p_3)\cdot p_4- (p_2 \times p_3)\cdot p_1\right) \end{aligned}$$

According to Eq. (1), we have to calculate $\nabla _{p_1}V,\nabla _{p_2}V,\nabla _{p_3}V$ and $\nabla _{p_4}V$ respectively. In order to make the derivation clearly, the basic gradient principles of a combination of cross and dot product can be represented as follows, where $a,b,c \in R^3$

4 $$\begin{aligned} \nabla _c(a \times b)\cdot c=\, & {} \nabla _c\left( (a_2b_3-a_3b_2)c_1 + (a_3b_1-a_1b_3)c_2 +(a_1b_2-a_2b_1)c_3\right) \nonumber \\=\, & {} a \times b,\end{aligned}$$

5 $$\begin{aligned} \nabla _c(a \times c)\cdot b=\, & {} \nabla _c\left( (a_2c_3-a_3c_2)b_1 + (a_3c_1-a_1c_3)b_2 +(a_1c_2-a_2c_1)b_3\right) \nonumber \\=\, & {} b \times a,\end{aligned}$$

6 $$\begin{aligned} \nabla _c(c \times a)\cdot b=\, & {} \nabla _c\left( (c_2a_3-c_3a_2)b_1 + (c_3a_1-c_1a_3)b_2 +(c_1a_2-c_2a_1)b_3\right) \nonumber \\=\, & {} a \times b. \end{aligned}$$

With the principles (4)–(6), we can deduce the corresponding gradient of Eq. (3) easily

7 $$\begin{aligned} \nabla _{p_1}V=\, & {} \frac{1}{6}(p_2 \times p_4 - p_3 \times p_4 - p_2 \times p_3) \nonumber \\=\, & {} \frac{1}{6}(p_2 \times p_4 - p_3 \times p_4 - p_2 \times p_3 - p_2 \times p_2) \nonumber \\=\, & {} \frac{1}{6}((p_4 - p_2)\times (p_3 - p_2)) \end{aligned}$$

Similarly, the gradient of other corners can be obtained

$$\begin{aligned} \nabla _{p_2}V=\, & {} \frac{1}{6}\left( (p_3-p_1)\times (p_4-p_1)\right) \\ \nabla _{p_3}V=\, & {} \frac{1}{6}\left( (p_4-p_1)\times (p_2-p_1)\right) \\ \nabla _{p_4}V=\, & {} \frac{1}{6}\left( (p_2-p_1)\times (p_3-p_1)\right) \end{aligned}$$

Then according to the above equations and Eqs. (4)–(7), the denominator in Eq. (1) can be represented as

8 $$\begin{aligned} \sum ^4_{j=1}w_j|\nabla p_j C(p)|^2 &= w_1\left| \frac{1}{6}(p_4-p_2)\times (p_3 -p_2)\right| ^2 + w_2\left| \frac{1}{6}(p_3-p_1)\times (p_4 -p_1)\right| ^2 \nonumber \\&\quad + w_3\left| \frac{1}{6}(p_4-p_1)\times (p_2 -p_1)\right| ^2 + w_4\left| \frac{1}{6}(p_2-p_1)\times (p_3 -p_1)\right| ^2 \end{aligned}$$

Now we have all the necessary factors in Eq. (1), which also means the desired volume conservation constraint function is achieved. With the volume conservation constraint mentioned above, realistic deformation result of a surgery training system can be improved greatly and our detailed experimental results will be given in the section of results and discussion.

### Hash table based spatial acceleration

The blood vessel model in a surgery training system usually has a large amount of tetrahedral elements, which make the vascular deformation calculation with volume conservation constraint much more time-consuming and may further limit the real-time performance of the system seriously. To guarantee a high real-time performance without losing deformation accuracy, several advanced algorithms and efficient data structures can be used. Inspired by the previous related work [21], a hash table based spatial acceleration algorithm is employed in our study to reduce computation overhead so as to significantly improve the real-time performance of our surgery training system. However, the original approach is used to process rapid collision detection between soft bodies, while in this paper we mainly use the hash value to quickly determine the vascular deformation region and then perform partial calculation which can improve real-time performance greatly.

**Fig. 3:**
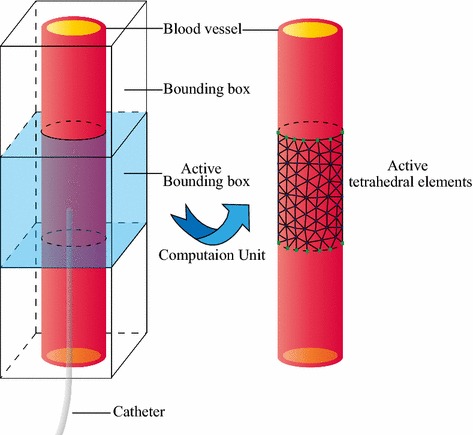
Illustration of the spatial acceleration approach. The bounding box *filled* with *light blue color* describes the active computation unit

Calculating vascular deformation partially is the core idea of the spatial acceleration approach. Figure 3 depicts the spatial acceleration approach with a small piece of blood vessel model, in which the wireframe and the filled box represent the inactive and active bounding boxes respectively. In the initialization stage, all tetrahedral elements of a blood vessel model are first classified into several adjacent child bounding boxes whose states are inactive by default and each accompanies with a unique hash value. The hash value is extremely important since it determines which bounding box encloses the catheter tip. As we mentioned above, a catheter can move freely in the blood vessel under the control of a skilled surgeon, thus a hash value of the catheter tip need to be calculated in every time-step. A bounding box will be activated when its hash value matches the hash value of the catheter tip, namely the active bounding box encloses the catheter tip. And further, all tetrahedral elements in the active bounding box are also active and will be used for deformation calculation, which means that an active bounding box is a basic calculation unit in our implementation.

During the simulation, some particles are fixed to prevent the blood vessel from falling under the gravity and moving freely under the pulled force. In our study, the particles which are too close to the top or bottom of the active bounding box are fixed. However, the catheter moves from one bounding box to another dynamically and when the catheter tip just enters a new bounding box with small displacement, the vascular deformation can hardly occur because of the fixed particles. To address this issue and guarantee more smooth transition when the catheter tip moves from one bounding box to another, we expanded the child bounding box a little bigger than the regular one when classifying the tetrahedral elements into a specific child bounding box. As a result, a tetrahedral element may be existed in several adjacent bounding boxes at the same time because of the shared space. The larger the shared space is, the smoother the transition results can be obtained while the more computation overhead is required. The detailed hash table based spatial acceleration approach will be discussed below.

The entire simulation domain is enclosed by an axis-aligned bounding box (AABB) with $g^{max}$ and $g^{min}$ denoting its corresponding minimum and maximum coordinates, then the entire bounding box should be partitioned into several regular child bounding boxes with size *d*. Each bounding box accompanies with a hash value which stored in an array, the length of the array is $N_{array} = N_x \cdot N_y \cdot N_z$, and

9 $$\begin{aligned} (N_x, N_y, N_z) = \biggl (\biggl \lceil \frac{g^{max}_x-g^{min}_x}{d} \biggr \rceil , \biggl \lceil \frac{g^{max}_y-g^{min}_y}{d} \biggr \rceil , \biggl \lceil \frac{g^{max}_z-g^{min}_z}{d} \biggr \rceil \biggr ) \end{aligned}$$

where $N_x, N_y$ and $N_z$ denote the number of bounding boxes in the three perpendicular axes respectively. Once given a specific bounding box with three axis-based indices (*i*, *j*, *k*), we can use the following function *T* (*i*, *j*, *k*) to uniquely map the three-dimensional bounding box into an element of the continuous one-dimensional array.

10 $$\begin{aligned} T(i,j,k) = i + j\times N_x + k\times N_x \cdot N_y, (0,0,0) \le (i,j,k) \le (N_x,N_y,N_z) \end{aligned}$$

In function *T* (*i*, *j*, *k*), a triple loop is used to map the bounding box into the array one by one continuously without overlapping. And the regular child bounding box can be calculated with

11 $$\begin{aligned} \left\{ \begin{array}{lll} e^{min}(i,j,k) &{}=&{} g^{min}+d\times (i,j,k) \\ e^{max}(i,j,k) &{}=&{} e^{min}(i,j,k)+(d,d,d) \end{array} \right. \end{aligned}$$

According to the reference [21], the corresponding hash value of a bounding box can be determined with

12 $$\begin{aligned} h(T(i,j,k))=\bigl [(i\times u)\oplus (j\times v)\oplus (k\times w)\bigr ] \ mod \ N_{array} \end{aligned}$$

Here *u*, *v* and *w* are three large prime numbers to ensure a unique hash value for each bounding box.

Since a regular child bounding box is designed to be a basic computation unit, we have to determine all the tetrahedral elements that a regular child bounding box contains. Each regular child bounding box is expanded with the size $span_x=span_y=span_z=d/3$ to guarantee a much more smooth transition during the simulation. The elongation *span* is an experienced value and it can balance computation efficiency and visual deformation result quite well.

During the simulation, the catheter moves in the blood vessel model freely and the position of the catheter tip is $(x_t,y_t,z_t)$ at a given time t and then we can calculate the corresponding hash value with

13 $$\begin{aligned} h_{Tip} = \biggl [\biggl (\biggl \lfloor {\frac{x_t-g^{min}_x}{d}}\biggl \rfloor \times u\biggr ) \! \oplus \! \biggl (\biggl \lfloor {\frac{y_t-g^{min}_y}{d}}\biggl \rfloor \times v\biggr ) \! \oplus \! \biggl (\biggl \lfloor {\frac{z_t-g^{min}_z}{d}}\biggl \rfloor \times w\biggr )\biggr ] \ mod \ N_{array} \end{aligned}$$

The parameters *u*, *v* and *w* have the same meaning and value as in Eq. (12). Then the child bounding box with the same hash value of the catheter tip will be activated and used for precise collision detection and deformation calculation.

## Results and discussion

In this section, a systematic and comprehensive description of our verification experiments is given, which not only describes the performance of our proposed method, but also compares the proposed method with previous related methods properly. All the experiments were performed on a laptop with Intel Core i5 3230M, 4G RAM and GeForce 630M graphic card. Tetrahedral mesh was used in our study since it is necessary for the implementation of the following finite element method. There are many available tools to generate tetrahedral mesh from surface mesh, what we choose is TetGen [22], an efficient tetrahedral mesh generator. The whole geometric model has been partitioned into 80 continuous bounding boxes for further spatial acceleration. For simplification, we only concern the blood vessel branch that the catheter will enter in our experiments, and there are 3954 vertices and 12,434 tetrahedral elements in total after tetrahedralization.

**Fig. 4:**
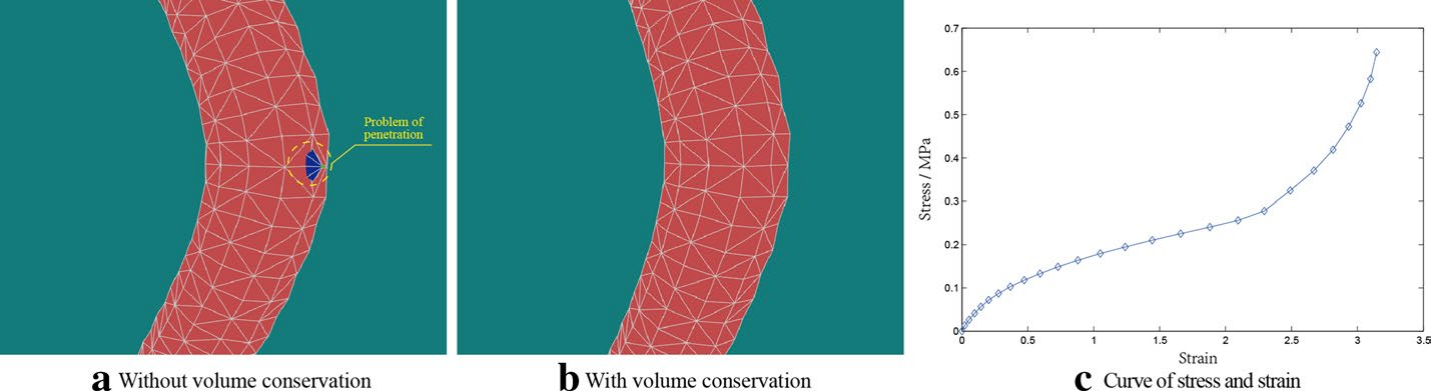
Visual deformation results and stress–strain curve using position-based dynamic approach. **a** A vertex in inner vascular wall has penetrated the outer vascular wall without volume conservation **b** a much more reliable result can be obtained with volume conservation **c** stress–strain curve of the collision vertex

In general position-based approach without the constraint of volume conservation, tetrahedral elements of a vascular model are much easier to penetrate each other during deformation if the loaded force reaches a certain threshold. When one or more vertices in the inner vascular wall penetrates the blood vessel, an erroneous visual result will be definitely obtained, as shown in Fig. 4a. We can see the problem of penetration from the figure clearly, where tetrahedral elements and vertices in the inner wall were drawn in blue and green color respectively. To build a robust interventional surgery training system, the problem of penetration is unacceptable, and as we mentioned above, volume conservation constraint is regarded as a promising way to address this kind of problem. The final visual rendering result of the improved position-based approach with the constraint of volume conservation is shown in Fig. 4b, which has addressed the penetration issue successfully. From these two figures, a conclusion that the employment of volume conservation constraint in position-based dynamic method can improve the visual deformation result greatly. Since reliable soft tissue deformation is important in a surgery training system, therefore, we have been attempting to use support vector machine method to refine the material parameters setting in our deformation modeling method [23]. In the study of virtual surgery simulation, the stress–strain curve is an essential criterion to assure that deformation is real and accurate. Therefore, we have also recorded a series of external force that applied to the collision vertex on the inner vascular wall and its corresponding displacement in the experiment, then we convert the contact force *F* to the stress with $\sigma =F / A$, where *A* is the area of the cross-section and the displacement into strain with $\varepsilon =(l-L) / L$, where *l* and *L* represent the current and original length of the fiber respectively. Finally a stress–strain curve can be obtained as shown in Fig. 4c, in which, we can see that the modeled blood vessel deforms a little when the contact force reaches a proper value and this phenomenon suits the non-linear property of soft tissue properly. From the figure, a conclusion that our deformation modeling method represents the biomechanics properties of soft tissue properly, can be obtained.

**Fig. 5:**
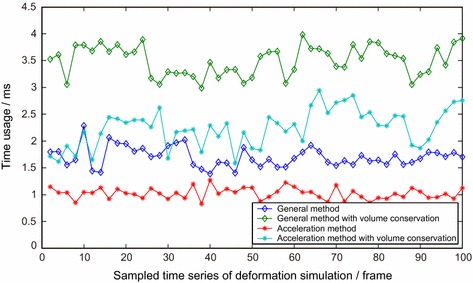
Real-time performance of the general and the acceleration methods. *Curves with* *diamond* and *asterisk* describe the time usage of the general and the acceleration methods respectively, the *green* and the *light blue curves* represent the position-based dynamic method with volume conservation constraint

Besides reliable deformation, computation efficiency is also an important factor in surgery training system since it determines the real-time performance of the system directly. However, computation overhead of our surgery training system increases significantly due to the employment of volume conservation constraint. In order to maintain a real-time deformation result in this case, hash table based spatial acceleration algorithm has been used to seek an optimal trade-off between efficiency and accuracy. Several necessary experiments with general or accelerated method were conducted to validate the computation efficiency of our approach, and each experiment was performed with and without volume conservation constraint respectively. The corresponding experimental results are shown in Fig. 5. From the figure, we can see that the deformation modeling method with volume conservation constraint is much more time-consuming than the general one, and the spatial acceleration method can reduce the computation time greatly and guarantee a better real-time performance as well. From Figs. 4 and 5, we can see that our acceleration method with volume conservation constraint can balance reliable deformation and real-time performance properly.

**Fig. 6:**
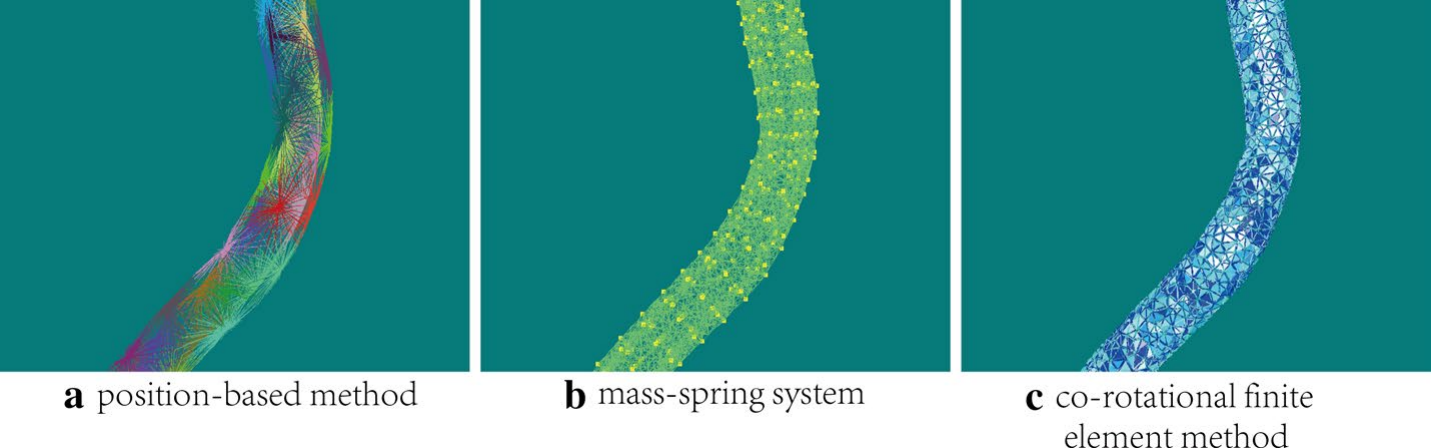
Illustration of three different implemented soft tissue deformation modeling methods with the same geometrical blood vessel model. **a** The force model of position-based method, **b** and **c** the force model of mass-spring system and finite element method respectively

There were already several feasible deformation modeling methods, in order to compare their performance with our method in this paper comprehensively, we have also implemented another two widely used methods, namely mass–spring system and co-rotational finite elements method, and their force model are shown in Fig. 6. Similar deformation results can be obtained using these methods with proper material parameters setting, but their efficiencies vary a lot. To compare their computation efficiencies, several key configurations like geometric model (tetrahedral vascular mesh) and numerical solver (implicit backward euler solver with time-step 0.02 s) are the same. Figure 6a illustrates the position-based approach with 64 clusters, and its average rendering speed is up to 116 frames per second which meets the requirement of real-time virtual surgery training system well. Figure 6b represents a mass-spring system based deformable blood vessel, where particles and springs were drawn in yellow and green respectively. Since each edge of the tetrahedral model is treated as an elastic spring in our implementation, and thus there are about 22K springs in total. As a result, the final rendering speed reduces to nearly 65 frames per seconds due to the large number of geometric primitives in the model. Lastly, Fig. 6c shows the force model of a more accurate co-rotational finite element method without any acceleration technique, and its average rendering speed as low as 12 frames per second which determines that this method cannot be employed in real-time surgery training system directly. As we mentioned above, the hybrid geometric blood vessel model processes collision detection and deformation calculation separately and hash table based spatial acceleration method make the partial deformation become possible, all these advantages can improve the real-time performance of our surgery training system.

**Fig. 7:**
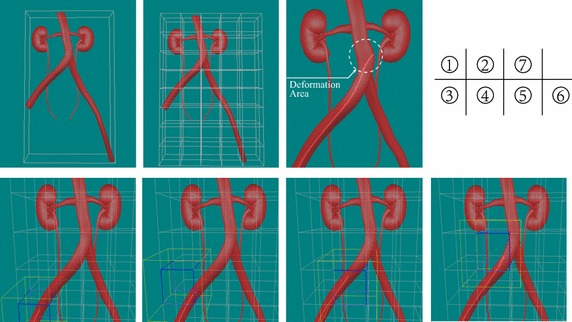
Visual results of our vascular interventional surgery training system. The *1st and 2nd figures* simply depict the global bounding box of the whole simulation domain and regular child bounding boxes after classification. The *figures from 3rd to 6th* describe the simulation process, where the *blue* and *yellow boxes* depict the activated child bounding box and its corresponding extended bounding box respectively. The *7th figure* shows the visual deformation result after detecting a collision in our system

**Fig. 8:**
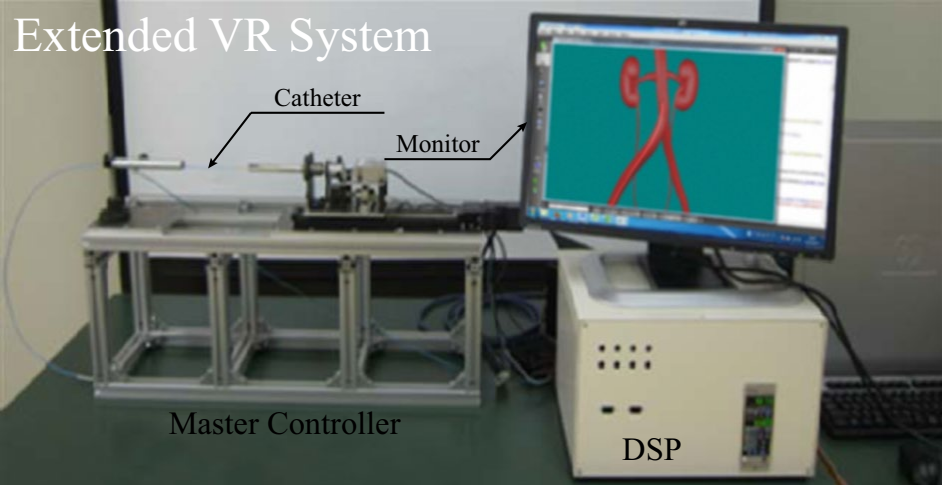
Snapshot of the entire surgery training system

In the final implementation of our real-time vascular interventional surgery simulator, besides efficient hybrid blood vessel model, position-based deformation with volume conservation and hash table based spatial acceleration were obtained. The final visual rendering results and system snapshot of our simulator are shown in Figs. 7 and 8 respectively. With video information provided by a monitor on the master side, an interventional radiologist operates the catheter along the main axis with the combination of translations and rotations. A series of continuous control instructions are transmitted to computer directly, once receiving the specific instructions, the catheter in the training system starts to follow the catheter in the master side. In addition, we have also invited two experienced surgeons from our partner hospital to perform a face validity of our surgery training simulator and their assessment mainly focus on three aspects, namely visual authenticity, haptic reality and user interaction, in which, the average score of visual authenticity is 0.78 (1.0 in total, the same as below) and they suggested that texture mapping technique can be used in the next version to improve the rendering results of the simulator. Besides, the average score of haptic reality and user interaction are 0.86 and 0.90 respectively which perform well during the evaluation and they thought that the simulator can simulate basic interventional operations well and its vascular deformation results are reliable, which should be helpful in clinic training and surgery scheme planning.

## Conclusions

Vascular interventional surgery is rapid developing and will let more and more patients benefit from it. In recent years, vascular interventional robotic technique has been built but still need to be refined. With the development of such a new coming useful technology, efficient surgeon training measures are still a big challenge. In order to design a reusable training platform, many scientists and researchers have paid lots of attention on this subject. In this paper, we have adapted a new hybrid blood vessel model for efficient collision detection and deformation calculation, which makes the movement of a catheter in the blood vessel more realistic. In addition, a spatial adaptive acceleration algorithm is employed to improve the real-time performance and visual deformation result, and the experimental results show that the hybrid blood vessel model and hash table based spatial adaptive acceleration approach are robust and efficient to guarantee the performance of our vascular surgery training system.

In future study, we will focus on the realistic material behavior of the blood vessel, such as non-linear, stress relaxation, anisotropy and so on. By employing the more realistic material properties, the deformation can be modeled much closer to the real blood vessel under the same external force load. Finally, we can use the training system to assist remote vascular interventional surgery.
